# Time trends in service provision and survival outcomes for patients with renal cancer treated by nephrectomy in England 2000–2010

**DOI:** 10.1111/bju.14217

**Published:** 2018-04-20

**Authors:** Ray C. J. Hsu, Matthew Barclay, Molly A. Loughran, Georgios Lyratzopoulos, Vincent J. Gnanapragasam, James N. Armitage

**Affiliations:** ^1^ Academic Urology Group Department of Surgery University of Cambridge Cambridge UK; ^2^ The Healthcare Improvement Studies (THIS) Institute University of Cambridge Cambridge UK; ^3^ Department of Urology Addenbrooke's Hospital Cambridge University Hospitals NHS Foundation Trust Cambridge UK; ^4^ Transforming Cancer Services Team National Health Service London UK; ^5^ National Cancer Registration and Analysis Service Public Health England London UK; ^6^ Epidemiology of Cancer Healthcare and Outcomes (ECHO) Group Department of Behavioural Science and Health University College London London UK

**Keywords:** #KidneyCancer, centralisation, nephrectomy, postoperative outcomes, renal cancer, survival

## Abstract

**Objective:**

To describe the temporal trends in nephrectomy practice and outcomes for English patients with renal cell carcinoma (RCC).

**Patients and Methods:**

Adult RCC nephrectomy patients treated between 2000 and 2010 were identified in the National Cancer Data Repository and Hospital Episode Statistics, and followed‐up until date of death or 31 December 2015 (*n* = 30 763). We estimated the annual frequency for each nephrectomy type, the hospital and surgeon numbers and their case volumes. We analysed short‐term surgical outcomes, as well as 1‐ and 5‐year relative survivals.

**Results:**

Annual RCC nephrectomy number increased by 66% during the study period. Hospital number decreased by 24%, whilst the median annual hospital volume increased from 10 to 23 (*P* < 0.01). Surgeon number increased by 27% (*P* < 0.01), doubling the median consultant number per hospital. The proportion of minimally invasive surgery (MIS) nephrectomies rose from 1% to 46%, whilst the proportion of nephron‐sparing surgeries (NSS) increased from 5% to 16%, with 29% of all T1 disease treated with partial nephrectomy in 2010 (*P* < 0.01). The 30‐day mortality rate halved from 2.4% to 1.1% and 90‐day mortality decreased from 4.9% to 2.6% (*P* < 0.01). The 1‐year relative survival rate increased from 86.9% to 93.4%, whilst the 5‐year relative survival rate rose from 68.2% to 81.2% (*P* < 0.01). Improvements were most notable in patients aged ≥65 years and those with T3 and T4 disease.

**Conclusions:**

Surgical RCC management has changed considerably with nephrectomy centralisation and increased NSS and MIS. In parallel, we observed significant improvements in short‐ and long‐term survival particularly for elderly patients and those with locally advanced disease.

## Introduction

Incidence of RCC has been increasing worldwide year‐on‐year by 3–4% and accounts for ~3% of all new cancer diagnoses in the UK 1, 2. Historically, RCC survival in England has lagged behind many other European countries 3, 4. Initiatives to improve outcomes through surgical service centralisation, reducing diagnostic delay, and increasing multidisciplinary team involvements in patient care were introduced in the *Improving outcomes in urological cancer* Guidance in 2002 5. In parallel, there is a growing move towards the use of nephron‐sparing surgery (NSS) and laparoscopic or robotic nephrectomy 6, 7. It is not currently known how adoption of these guidelines and practices has impacted patient outcomes, particularly on a nationwide level. Many population‐based studies examining RCC survival have focused on patients irrespective of treatment modality and may include those who received no active treatment 8, 9, 10. Interpretation of findings from these studies is however challenging, as nephrectomy is often considered the only potentially curative treatment for RCC and inclusion of other treatment modalities may dilute the specific effect of changes in surgical practice. In the present study, we describe the changes to RCC nephrectomy activity and service provision in England in recent years and investigate temporal trends in short‐ and long‐term outcomes with a particular focus on survival.

## Patients and methods

### Data

Data extract was supplied by Public Health England National Cancer Registration and Analysis Service (NCRAS) and contains fields from both the National Cancer Data Repository (NCDR) and Hospital Episode Statistics (HES) Admitted Patient Care. The NCDR contains tumour level records submitted by the eight English Cancer Registries, together with survival information sourced from the Office for National Statistics (ONS) 11. HES is a major administrative database containing details of all patients admitted to NHS hospitals in England 12. Together, hospital, patient, and tumour level information can be directly extracted or derived from the supplied linked database. Individual data fields and their source are described in Table S1. Ethics approval was granted by the National Research Ethics Committee (reference 15/EM/0340) and Confidentiality Advisory Group (reference 15/CAG/0169).

### Exposure variables

We included all patients diagnosed with RCC and treated with either radical or partial nephrectomy between 2000 and 2010, regardless of tumour stage. We excluded those aged ≤17 years at the time of surgery, as well as those diagnosed with upper tract urothelial carcinoma. Patients treated with nephroureterectomy, nephrectomy of transplanted kidney or focal therapies were also excluded from our study population. The cohort was identified using the International Statistical Classification of Diseases and Related Health Problems 10th revision (ICD10) code and Office of Population Censuses and Surveys Classification of Interventions and Procedures version 4 (OPCS4) code (Table S2).

Type of nephrectomy and surgical access were derived from HES procedure codes. Operative caseloads were calculated based on the number of nephrectomies performed per annum by the responsible hospital as recorded in HES. Within the NHS, a Trust may manage more than one local hospital. In our analysis, we treat all hospitals within the same Trust as a single organisation. Similarly, we calculated surgeons’ operative caseloads using the same method. To account for potential coding errors of the responsible clinician, we only included surgeons recorded as having performed five or more nephrectomies per year.

Patient demographics including age, sex, ethnicity, and socioeconomic deprivation together with admission details such as admission method and waiting time were extracted directly from the NCDR or HES. We tabulated patient comorbidities by using the Royal College of Surgeons Charlson Score 13. ICD10 diagnosis codes recorded in HES from the index nephrectomy admission and up to 1 year prior were used to estimate the number of comorbid conditions each patient had at the time of the operation. Diagnoses from the index hospital admission were excluded if they were likely to be complications of surgery rather than comorbid conditions.

### Outcome variables

We calculated the population nephrectomy rate by dividing the total number of nephrectomies in England per annum over the mid‐year English population estimate as published by the ONS 14. The total number of nephrectomies was compared with the number of RCCs registered in England to provide the proportion of RCCs resected each year 15.

Re‐admissions were identified if patients had a new admission within 30 days of discharge from the index nephrectomy admission and a concurrent emergency status recorded as the method of admission. Length of stay was calculated by taking the differences between date of nephrectomy and date of discharge.

Short‐term surgical outcomes including infection, haemorrhage and transfusion were derived from the ICD10 diagnosis fields in HES. Survival time was calculated from date of surgery until date of death or 31 December 2015. In‐hospital mortality was derived if patient was recorded as having died in the HES discharge method field.

### Analysis

We summarised time trend by calculating the annual average percent change (AAPC) across the study period using the Joinpoint Regression Program 16, 17. *P* values <0.05 were considered statistically significant. All other statistical analyses were performed using Stata Statistical Software: release 14 (StataCorp., College Station, TX, USA) 18.

We estimated relative survival, defined as the ratio of the observed survival in our cohort to the expected survival in the general population. It is an estimate of the survival rate that would be observed if patients could only die from their RCC and is analogous to cause‐specific survival. A major advantage of measuring relative survival is that cause of death, which may be unreliable in registry data, is not required. Relative survival rates were capped at 100%. We used English population life tables stratified by age, sex and calendar year to calculate expected survival rates and these were available online from the Cancer Survival Group at the London School of Hygiene and Tropical Medicine 19. We used rates from 2009 for years beyond 2009. Relative survival rates were estimated using the *strs* package in Stata 20. To avoid possible confounding caused by shifts in patients’ age profile, we estimated relative survival separately for age groups ≤64, 65–74, and ≥75 years, and standardised to the age distribution of our incident cohort in 2010. Amongst patients with observed stage, subgroup analyses stratified by T stage were performed on patients with localised disease, excluding those recorded as having nodal or distant metastases (N1 or M1 or above).

## Results

A total of 30 763 adult RCC nephrectomies were performed in England between 2000 and 2010, with a median follow‐up of 9.3 years. Data fields from HES were largely complete except for ethnicity and waiting time, recorded in 92.6% and 91.4% of the patients, respectively. Tumour level data from the NCDR were less complete with 41.4% of patients having tumour size recorded and 46.7% of patients with T stage data.

### Changes in nephrectomy activity

**Figure 1:**
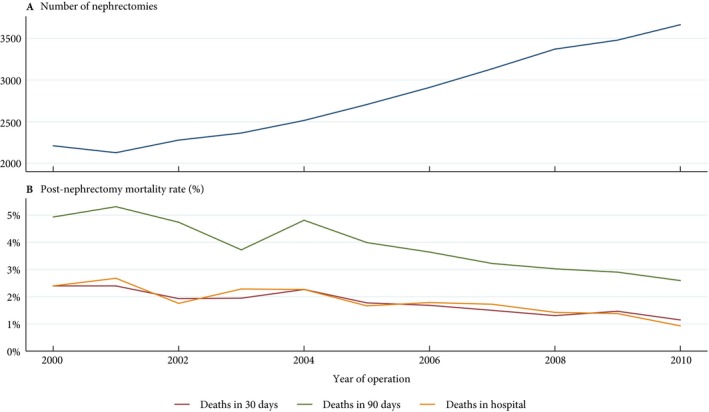
**A**. Total number of RCC nephrectomies performed in England and **B**. post‐nephrectomy deaths in each calendar year.

The annual number of nephrectomies in England increased overall by 65.7% from 2211 in 2000 to 3664 in 2010 (Fig. 1A). In the same time period, the crude annual nephrectomy rate in the general population increased from 4.5 to 7.0 per 100 000 people per year, an average annual increase of 4.9% (*P* < 0.01) (Table 1). Looking specifically at RCC registered in England, 50.5% of those diagnosed in 2000 had their cancer resected, increasing to a peak of 55.9% for those diagnosed in 2008. There appeared to be broadly similar increasing trends for men and women and for patients aged 18–64 and 65–74 years. Use of nephrectomy for patients aged ≥75 years appeared stable at ~30%.

**Table 1:** Number of nephrectomies and their types and approach in England between 2000 and 2010

	Year											AAPC, %	*P*
	2000	2001	2002	2003	2004	2005	2006	2007	2008	2009	2010		
Nephrectomy patients, *n*	2211	2128	2279	2364	2515	2706	2911	3135	3371	3479	3664		
Nephrectomy rate (per 100 000 people)	4.5	4.3	4.6	4.7	5.0	5.3	5.7	6.1	6.5	6.7	7.0	4.9	<0.01
%:													
Proportion of RCCs resected (by year of diagnosis)	50.5	49.3	49.6	50.3	51.2	52.9	52.9	54.6	55.9	53.9	50.9	0.5	0.36
Male	51.5	51.0	50.7	51.6	52.8	53.0	52.6	55.1	56.1	55.5	50.7	0.3	0.56
Female	48.9	46.4	47.7	48.2	48.6	52.8	53.5	53.7	55.6	51.5	51.2	0.9	0.39
Age, years													
<65	64.7	65.0	62.7	68.2	67.6	68.9	70.1	68.7	72.9	70.6	66.7	0.9	0.02
65–74	53.6	53.2	53.4	53.0	56.0	56.6	58.9	60.3	61.6	57.4	55.8	0.5	0.40
≥75	30.0	27.0	29.5	26.1	26.8	30.1	28.7	31.2	30.7	30.1	27.6	0.5	0.41
Nephrectomy type, *n* (%)													
Radical	2111 (95.5)	2040 (95.9)	2158 (94.7)	2216 (93.7)	2324 (92.4)	2488 (91.9)	2641 (90.7)	2832(90.3)	2997 (88.9)	3035 (87.2)	3078 (84.0)	–1.3	<0.01
Partial	105 (4.8)	93 (4.4)	124 (5.4)	149 (6.3)	197 (7.8)	223 (8.2)	270 (9.3)	308 (9.8)	379 (11.2)	455 (13.1)	589 (16.1)	13.2	<0.01
T1, *n* (%^a^)	9 (6.4)	14 (7.3)	22 (8.7)	45 (14.5)	39 (10.9)	62 (14.7)	77 (14.9)	103 (15.5)	158 (17.8)	227 (23.1)	362 (29.0)	14.4	<0.01
T2, *n* (%^a^)	–	1 (0.7)	4 (2.3)	4 (2.0)	8 (4.0)	5 (2.1)	5 (2.0)	5 (1.7)	6 (1.8)	11 (3.3)	15 (3.6)	8.9	0.12
Type of access, *n* (%)													
Open	2198 (99.4)	2081 (97.8)	2171 (95.3)	2160 (91.4)	2175 (86.5)	2166 (80.0)	2177 (74.8)	2124 (67.8)	2138 (63.4)	2056 (59.1)	1964 (53.6)	–5.9	<0.01
Laparoscopic	13 (0.6)	47 (2.2)	108 (4.7)	204 (8.6)	340 (13.5)	540 (20.0)	734 (25.2)	1011 (32.3)	1228 (36.4)	1413 (40.6)	1663 (45.4)	54.6	<0.01
Robot‐assisted	–	–	–	–	–	–	–	–	5 (0.2)	10 (0.3)	37 (1.0)		

a Denotes proportion of RCC performed under partial nephrectomy in patients with identical T stage.

Partial nephrectomy was increasingly utilised, accounting for 16.1% of all RCC surgery and 29.0% of all T1 disease by the end of the study (*P* < 0.01). Use of minimally invasive surgery (MIS) increased to 46.4% of all RCC nephrectomies by 2010 (*P* < 0.01).

### Changes in service provision

The number of hospitals performing any RCC nephrectomy decreased by one‐quarter over the study period, an average reduction of 2.8% per annum (*P* < 0.01) (Table 2). This decline was only seen for hospitals performing radical nephrectomy, whilst the number of hospitals performing partial nephrectomy increased on average by 5.9% per year (*P* < 0.01). The number of hospitals performing MIS nephrectomy also increased, with 118 of the 136 (86.8%) hospitals performing any nephrectomy in 2010 adopting the technique. The median hospital nephrectomy volume increased from 10 per hospital in 2000 to 23 per hospital in 2010 (*P* < 0.01), with median partial nephrectomy volume increasing from one to five (*P* < 0.01) and laparoscopic or robot‐assisted nephrectomy volume increasing from one to 12 (*P* < 0.01).

**Table 2:** Changes to hospital and surgeon nephrectomy activities in England between 2000 and 2010

	Year											AAPC, %	*P*
	2000	2001	2002	2003	2004	2005	2006	2007	2008	2009	2010		
Hospitals													
Performing nephrectomy (any), *n*	180	179	168	156	149	148	145	146	141	143	136	–2.8	<0.01
Performing RN, *n*	180	179	168	155	149	148	145	145	139	143	136	–2.8	<0.01
Performing PN, *n*	56	51	61	63	69	77	80	81	86	88	91	5.9	<0.01
Performing MIS nephrectomy, *n*	13	22	32	49	68	86	100	103	112	117	118	23.6	<0.01
Nephrectomy volume, *n* median (IQR)	10 (6–15)	10 (5–15)	11.5 (7–18)	13 (7.5–20.5)	13 (8–22)	14.5 (9–23.5)	17 (11–25)	17 (10–29)	20 (12–32)	23 (10–33)	23 (12–39.5)	9.5	<0.01
RN volume, *n* Median (IQR)	10 (6–14)	10 (5–15)	11 (6–17)	12 (7–19)	13 (8–20)	14 (9–21)	15 (10–23)	16 (10–26)	18 (11–29)	20 (10–30)	20 (11.5–32)	8.0	<0.01
PN volume, *n*, Median (IQR)	1 (1–2)	1 (1–2)	1 (1–3)	1 (1–3)	2 (1–3)	2 (1–3)	2 (1–4)	3 (1–4)	3 (2–5)	3 (1–7)	5 (2–9)	17.7	<0.01
MIS nephrectomy volume, *n* median (IQR)	1 (1–2)	1 (1–2)	2 (1–5)	3 (1–5)	3 (2–6)	4 (2–9)	5 (3–9)	7 (3–13)	8.5 (4–13.5)	10 (4–17)	12 (6–18)	29.2	<0.01
Surgeons													
Performing nephrectomy (any), *n*	191	181	183	192	203	204	220	214	235	252	242	3.3	<0.01
Performing RN, *n*	181	175	176	180	191	190	205	201	223	233	223	2.9	<0.01
Performing PN, *n*	1	3	5	4	6	10	13	12	18	33	39	37.1	<0.01
Performing MIS nephrectomy, *n*	0	3	7	11	22	37	57	71	89	111	128	51.8	<0.01
Nephrectomy volume, *n* median (IQR)	7 (6–9)	7 (5–9)	7 (6–10)	7 (6‐10)	7 (6–11)	8 (6–12)	8 (6–13)	9 (6–16)	10 (7–14)	10 (7–14)	10.5 (7–16)	4.6	<0.01
RN volume, *n* median (IQR)	7 (6–9)	7 (5–9)	7 (6–10)	7 (6–10)	7 (5–11)	8 (6–11)	8 (6–12)	9 (6–14)	9 (6–13)	9 (7–13)	10 (7–14)	3.5	<0.01
PN volume, *n* median (IQR)	5 (5–5)	5 (5–5)	5 (5–5)	5 (5–6)	6 (5–6)	7 (6–7)	7 (6–12)	10 (7–13.5)	7 (5–12)	7 (5.5–10)	8 (5–11)	5.8	<0.01
MIS nephrectomy volume, *n* median (IQR)	0	6 (5–6)	6 (5–9)	8 (6–10)	6 (5.5–8)	9 (6–10)	7 (5–10)	8 (6.5–12)	9 (7–12)	8.5 (7–11)	9 (6‐13)	4.9	<0.01
Consultants/hospital, *n* median (IQR)	1 (0–2)	1 (0–2)	1 (1–2)	1 (0–2)	1 (1–2)	1 (1–2)	1 (1–2)	1.5 (1–2)	2 (1–2)	2 (1–3)	2 (1–3)	8.7	<0.01

RN, radical nephrectomy; PN, partial nephrectomy.

The number of surgeons performing RCC nephrectomy increased overall regardless of the type of nephrectomy or access (*P* < 0.01) (Table 2). The most pronounced increase was seen for surgeons performing MIS nephrectomy, with an average increase of 51.8% per annum (*P* < 0.01). Consultant teams also expanded with the median number of surgeons per hospital increasing two‐fold over the study period (*P* < 0.01). The median nephrectomy volume per surgeon grew from 7 to 10.5 (*P* < 0.01). Similar growths were seen across different types of nephrectomy and approach.

Despite the year‐on‐year growth in the number of nephrectomies performed in England, we observed a reduction in the number of nephrectomy‐performing hospitals and an increase in the number of nephrectomy‐performing surgeons, with paralleled increase in both hospital and surgeon nephrectomy volumes, suggestive of service centralisation.

### Changes in patient characteristics and outcomes

The mean age and sex distribution remained stable during the 11‐year period and the proportion of White patients decreased from 95.7% to 94.1% (*P* < 0.01) (Table S3). The proportion of patients with multiple recorded comorbidities increased significantly and by 2010, 46.8% of RCC nephrectomy patients had one or more recorded comorbidities (*P* < 0.01).

Of those patients with available tumour data, the mean tumour size decreased from 71.5 to 64.5 mm (*P* < 0.01). An increasing proportion of nephrectomies involved smaller tumours (T1) and localised disease (Stage I) accounting for 46.2% and 39.0%, respectively, in 2010 (*P* < 0.01).

The median time patients waited for their operation increased overall (*P* = 0.01), and appears to have been stable between 22 and 25 days since 2003 (Table 3). The proportion of nephrectomies performed under emergency admission decreased from 9.0% to a low of 3.0% in 2010 (*P* < 0.01). The median length of stay decreased regardless of the type of nephrectomy or approach, reducing overall from 8 days in 2000 to 5 days in 2010 (*P* < 0.01). However, the percentage of patients re‐admitted as an emergency within 30 days of discharge rose from 8.1% to 10.5% (*P* < 0.01). There was no significant change in postoperative infection or haemorrhage rates, but a significant reduction in transfusion rates was noted, with only 0.4% of patients in 2010 requiring transfusion compared to 6.6% in 2000 (*P* < 0.01).

**Table 3:** Outcomes of RCC nephrectomy patients by year of operation

	Year												AAPC, %	*P*
	*N*	2000	2001	2002	2003	2004	2005	2006	2007	2008	2009	2010		
Waiting time, days, median (IQR)	28130	19 (10–32)	19 (10–32)	21 (12–37)	22 (12–37)	24 (13–40)	25 (13–39)	22 (13–32)	22 (13–32)	22 (13–33)	24 (14–35)	25 (15–36)	2.2	0.01
Emergency admission, *n* (%)	30763	198 (9.0)	146 (6.9)	157 (6.9)	146 (6.2)	148 (5.9)	157 (5.8)	141 (4.8)	123 (3.9)	137 (4.1)	119 (3.4)	110 (3.0)	–9.5	<0.01
LOS, days, median (IQR)	30719	8 (6–10)	8 (6–11)	7 (6–10)	7 (6–10)	7 (6–10)	7 (5–9)	6 (5–9)	6 (4–8)	6 (4–8)	6 (4–8)	5 (4–7)	–3.9	<0.01
Open		8 (6–10)	8 (6–11)	7 (6–10)	7 (6–10)	8 (6–11)	7 (6–10)	7 (5–9)	7 (5–9)	7 (5–9)	7 (5–9)	6 (5–9)	–1.9	0.01
MIS		7 (6–10)	5 (4–7)	5 (4–7.5)	4 (3–7)	5 (3–6)	5 (3–6)	4 (3–6)	4 (3–6)	4 (3–6)	4 (3–6)	4 (3–6)	–4.1	0.01
RN		8 (6–10)	8 (6–11)	7 (6–10)	7 (6–10)	7 (6–10)	7 (5–9)	6 (5–9)	6 (4–8)	6 (4–8)	6 (4–8)	5 (4–7)	–3.9	<0.01
PN		7 (6–10)	7 (6–9)	7 (6–10)	7 (6–10)	7 (6–9)	7 (5–9)	7 (5–9)	6 (5–8)	6 (4–8)	6 (4–8)	6 (4–7)	–1.9	<0.01
Readmission, *n* (%)	30763	180 (8.1)	153 (7.2)	197 (8.6)	195 (8.3)	262 (10.4)	246 (9.1)	268 (9.2)	318 (10.1)	307 (9.1)	355 (10.2)	384 (10.5)	2.9	<0.01
Infection, *n* (%)	30763	52 (2.4)	49 (2.3)	36 (1.6)	61 (2.6)	50 (2.0)	53 (2.0)	63 (2.2)	79 (2.5)	93 (2.8)	94 (2.7)	101 (2.8)	2.9	0.09
Haemorrhage, *n* (%)	30763	52 (2.4)	62 (2.9)	60 (2.6)	55 (2.3)	68 (2.7)	74 (2.7)	71 (2.4)	79 (2.5)	83 (2.5)	117 (3.4)	127 (3.5)	3.6	0.16
Transfusion, *n* (%)	30763	146 (6.6)	122 (5.7)	133 (5.8)	144 (6.1)	159 (6.3)	114 (4.2)	57 (2.0)	21 (0.7)	23 (0.7)	11 (0.3)	13 (0.4)	–27.7	<0.01
30‐day mortality, *n* (%)	30763	53 (2.4)	51 (2.4)	44 (1.9)	46 (1.9)	57 (2.3)	48 (1.8)	49 (1.7)	47 (1.5)	44 (1.3)	51 (1.5)	42 (1.1)	–6.8	<0.01
90‐day mortality, *n* (%)	30763	109 (4.9)	113 (5.3)	108 (4.7)	88 (3.7)	121 (4.8)	108 (4.0)	106 (3.6)	101 (3.2)	102 (3.0)	101 (2.9)	95 (2.6)	–6.6	<0.01
In‐hospital mortality, *n* (%)	30763	53 (2.4)	57 (2.7)	40 (1.8)	54 (2.3)	57 (2.3)	45 (1.7)	52 (1.8)	54 (1.7)	48 (1.4)	48 (1.4)	34 (0.9)	–8.0	<0.01

LOS, length of stay; RN, radical nephrectomy; PN, partial nephrectomy.

Figure 1B shows the trend in short‐term mortality throughout the study period. The 30‐day mortality rate decreased from 2.4% to 1.1% (*P* < 0.01). Similar reductions were seen for 90‐day mortality (p<0.01) and in‐hospital mortality (*P* < 0.01), which had an average year‐on‐year decrease of 6.6% and 8.0%, respectively. These were in keeping with the increase in 30‐ and 90‐day age‐standardised relative survival rates, which saw absolute increases of 1.1% and 2.2%, respectively (*P* < 0.01). The reduction in mortality was most prominent in patients aged ≥65 years regardless of the follow‐up period (Table S4).

**Figure 2:**
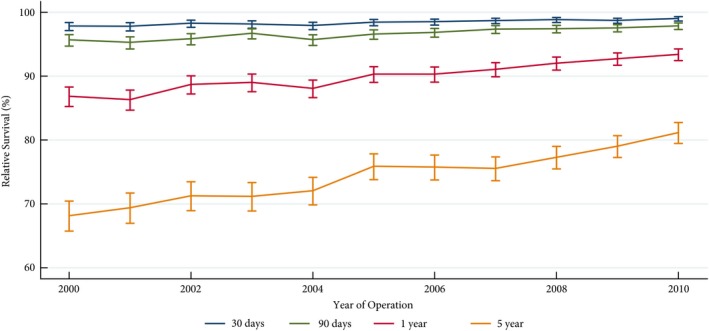
The 30‐day, 90‐day, 1‐year and 5‐year age‐adjusted relative survival rates in patients with RCC treated with nephrectomy between 2000 and 2010.

The 1‐ and 5‐year age‐standardised relative survival for all RCC nephrectomies increased to 93.4% and 81.2%, respectively by 2010, from 86.9% and 68.2%, respectively in 2000 (*P* < 0.01) (Fig. 2). Improvements in relative survival seemed primarily driven by patients with locally advanced disease (T3 and T4), for whom 1‐ and 5‐year relative survival increased from 83.9% to 91.8% (*P* < 0.01) and from 59.0% to 71.6%, respectively (*P* = 0.01) (Table 4).

**Table 4:** Age‐standardised 30‐day, 90‐day, 1‐year and 5‐year relative survival rates stratified by T stage for patients with localised RCC treated with nephrectomy

Relative survival rate	Year											AAPC, %	*P*
	2000	2001	2002	2003	2004	2005	2006	2007	2008	2009	2010		
T1 (*n* = 5828)													
30‐day % (95% CI)	99.1 (91.9–99.9)	99.7 (93.4–100)	99.8 (93.3–100)	99.2 (96.9–99.8)	100 (–)	99.6 (97.3–100)	99.6 (98.2–99.9)	100 (–)	99.5 (98.5–99.8)	99.6 (98.7–99.9)	99.7 (98.9–99.9)	0.03	0.35
90‐day % (95% CI)	97.9 (91.8–99.5)	99.6 (90.1–100)	99.7 (86.2–100)	98.9 (96.2–99.8)	100 (–)	99.7 (93.0–100)	99.6 (97.2–99.9)	100 (–)	99.1 (97.8–99.6)	99.5 (98.3–99.9)	99.7 (98.4–99.9)	0.1	0.17
1‐year % (95% CI)	99.5 (11.8–100)	99.7 (1.6–100)	97.7 (93.2–99.2)	98.7 (93.8–99.7)	99.7 (46.2–100)	98.7 (95.1–99.7)	98.2 (95.5–99.3)	99.6 (91.5–100)	99.1 (96.8–99.7)	98.9 (97.0–99.6)	99.3 (97.4–99.8)	<0.01	0.96
5‐year % (95% CI)	92.6 (79.6–97.5)	92.7 (83.8–96.8)	92.6 (84.9–96.5)	95.4 (87.6–98.3)	98.7 (77.5–99.9)	92.6 (87.1–95.8)	92.6 (87.9–95.5)	97.0 (91.8–98.9)	94.2 (90.7–96.4)	96.4 (92.8–98.2)	97.2 (93.9–98.7)	0.4	0.10
T2 (*n* = 2416)													
30‐day % (95% CI)	100 (–)	98.7 (94.0–99.7)	97.8 (93.8–99.2)	99.3 (96.0–99.9)	100 (–)	100 (–)	99.1 (95.7–99.8)	99.0 (96.3–99.7)	99.8 (93.9–100)	99.0 (96.5–99.7)	98.3 (96.3–99.2)	–0.03	0.74
90‐day % (95% CI)	100 (–)	99.1 (91.4–99.9)	96.9 (92.3–98.8)	99.2 (94.8–99.9)	100 (–)	99.5 (92.3–100)	99.4 (93.5–99.9)	98.1 (94.8–99.3)	99.0 (96.0–99.8)	98.6 (95.7–99.6)	97.5 (95.2–98.7)	–0.1	0.32
1‐year % (95% CI)	96.1 (86.9–98.9)	96.3 (88.7–98.8)	92.0 (85.9–95.6)	94.6 (88.3–97.5)	99.0 (86.2–99.9)	95.9 (91.1–98.1)	97.5 (92.4–99.2)	96.0 (91.6–98.1)	96.0 (92.1–98.0)	96.3 (92.4–98.2)	97.2 (94.1–98.7)	0.2	0.33
5‐year % (95% CI)	86.5 (73.3–93.5)	74.4 (63.1–82.7)	75.9 (66.6–82.9)	85.0 (75.1–91.1)	81.6 (71.9–88.2)	88.0 (79.9–92.9)	88.2 (80.2–93.1)	85.6 (78.3–90.5)	82.9 (76.3–87.8)	88.5 (81.7–92.9)	86.0 (81.7–92.9)	0.9	0.10
T3 (*n* = 4320) and T4 (*n* = 158)													
30‐day % (95% CI)	97.3 (93.3–98.9)	96.3 (92.4–98.3)	99.0 (96.2–99.7)	98.1 (95.8–99.2)	96.1 (93.2–97.8)	96.3 (93.8–97.8)	98.7 (96.5–99.5)	98.6 (96.9–99.3)	99.2 (98.1–99.7)	98.5 (97.2–99.2)	98.9 (97.8–99.5)	0.2	0.09
90‐day % (95% CI)	95.4 (90.6–97.8)	93.2 (88.4–96.1)	95.5 (91.8–97.6)	97.5 (94.8–98.8)	92.9 (89.4–95.2)	95.3 (92.5–97.1)	97.3 (94.5–98.7)	96.9 (94.7–98.2)	97.3 (95.5–98.4)	96.6 (94.9–97.8)	97.9 (96.5–98.8)	0.3	0.05
1‐year % (95% CI)	83.9 (76.8–89.0)	80.3 (73.5–85.5)	86.1 (80.6–90.1)	88.3 (83.8–91.7)	82.4 (77.5–86.4)	86.1 (81.8–89.5)	86.6 (82.0–90.0)	87.6 (83.9–90.5)	88.9 (85.8–91.4)	89.2 (86.4–91.5)	91.8 (89.4–93.7)	0.9	<0.01
5‐year % (95% CI)	59.0 (49.5–67.4)	51.2 (42.8–58.9)	65.9 (58.1–72.5)	60.6 (54.0–66.6)	59.9 (53.3–65.8)	67.6 (61.6–72.8)	66.7 (60.2–72.3)	66.3 (61.0–71.1)	63.8 (59.1–68.1)	68.6 (64.2–72.5)	71.6 (67.6–75.1)	2.1	0.01

## Discussion

Between 2000 and 2010 the annual number of RCC nephrectomies performed in England increased by nearly 66%, with a greater proportion of RCCs treated surgically. Nephrectomy centralisation is evident with increasing median case volumes for both hospitals and surgeons. There was also a rapid increase in the use of NSS and MIS. During this period, postoperative outcomes and long‐term survival rates showed significant trends of improvement.

Changes to nephrectomy practice are in part secondary to the shift in tumour stage distribution and the adoption of NSS as a standard for early stage disease. Increasing detection of small renal tumours have led to the increase in RCC incidence, reflected in the growing proportion of T1 disease in our present cohort 21. Partial nephrectomy is also increasingly the standard surgical treatment for T1 renal tumours 22 and emerging as an acceptable alternative for selected T2 disease 23. Patients previously not candidates for radical treatment due to risk of renal failure may now be considered for NSS, where further renal function deterioration is mitigated through the preservation of renal parenchyma 24.

We tracked the centralisation of RCC nephrectomy, reflecting similar trends in other established healthcare systems 25. However, we observed centralisation occurring across the board for all nephrectomy types, in contrast to recommendations that focused primarily on the 20% of RCC cases where patients present with bilateral disease, resectable metastatic disease, hereditary papillary or von Hippel‐Lindau disease, and for those suitable for NSS or whose tumours have invaded the large veins 5. This may be secondary to hospital mergers and the potential reduction in costs, and there also appears to be a trend of improving outcomes in nephrectomy centralisation 26, 27, 28

Whilst the use of NSS and MIS techniques has increased rapidly in the past decade, there may still be a delay in England in adopting surgical innovations. At its peak, partial nephrectomy was performed in 16% of all RCC nephrectomies, behind the 31–32% reported in other European and USA series over the same period 29, 30, 31. It is unclear whether stage distributions were comparable across the different series, but the number and proportion of partial nephrectomies estimated from our present data are broadly consistent to estimates from an earlier British study 32. The difference is even more remarkable when comparing only T1 disease, where 29% of patients in England were treated with NSS compared to 57% in a multinational multicentre study 33, although institutional data may not fully represent nationwide practice, particularly as hospital level factors can significantly influence the utilisation of partial nephrectomy 34.

Historical studies showed conflicting results for short‐term nephrectomy morbidity and mortality trends over time 26, 35. We found clear improvements in surgical mortality, with the 30‐day mortality rate decreasing to 1.1% in 2010, similar to the 0.9–2.7% reported by other countries during the same time period 36, 37, 38. The greatest improvements were seen in elderly patients aged ≥65 years, suggesting greater safety in surgery with reduced outcome inequality for patients of different ages and were consistent with the improvements seen in other urological procedures 39.

Long‐term post‐nephrectomy RCC outcomes have so far been poorly reported at national levels. We observed a substantial survival increase at 1 and 5 years after surgery, to levels similar to the cancer‐specific survival rates reported by other series for patients treated during the same periods, even when T stage is taken into consideration 40, 41. Advances in systemic therapy or other host factors, such as progressively fitter patients, may have contributed to the improvement. However, adjuvant or neoadjuvant therapy have not traditionally been advocated for patients with locally advanced disease, a group we observed the most significant survival increase, which would suggest that the improving trend is in part due to the improvements in surgical quality and care.

To the best of our knowledge, this is the first study using routinely collected data with whole population coverage to examine long‐term survival specifically in patients with RCC treated surgically. Results from the BAUS nephrectomy dataset have previously been published and reported on outcomes in periods beyond those covered in the present study 32. A similar number of RCC nephrectomies, proportion of NSS, length of stay, and 30‐day mortalities were observed between our estimates and the BAUS study, particularly when time trends are taken into consideration. However, the main advantage of our present study is the large, unselected sample with mandatory reporting. Case ascertainment from the English cancer registries and the NCDR has also been estimated to be >98%, compared to 80% for the BAUS dataset 42. In addition, the NCDR and HES provide data with long follow‐up duration, not currently available through the BAUS dataset, and capture the changing RCC landscape and trend in nephrectomy activities in England.

The limitations are most notably the inconsistency in coding, which may be dependent on clinical coders and subject to changes in government policy. Administrative data such as HES were initially established primarily for reimbursement purposes and therefore do not record clinical parameters that may be relevant to nephrectomy outcome, e.g. ischaemic time. We were also unable to identify and adjust for disease recurrence or use of systemic therapy, and these may have implications on intermediate‐ and long‐term outcomes. A significant proportion of stage data were missing in our present cohort and results should be interpreted taking this into consideration. Extensive work has led to improvements in data completeness and accuracy in the NCDR and HES, and use of these data therefore remains justified particularly when linkage between the two provides complementary information that compensates each other's shortcomings. We were also only able to analyse data from patients treated up to 2010 and this may not reflect contemporary practice.

Shift in disease stage, service centralisation, and both surgical and non‐surgical innovations, likely all contributed to the survival increase. Future studies should focus on quantifying each factor's influence on the improvements. Linkage of data between national and local specialty databases should provide additional clinical parameters for more comprehensive analyses, whilst maintaining the statistical power of large datasets. Results will be valuable for the continuous monitoring of RCC nephrectomy performance and allow minimum acceptable standards to be set for individual nephrectomy providers.

In this comprehensive analysis of national data, we found significant changes to RCC nephrectomy practice in England over the past decade, with nephrectomy centralisation and rapid adoption of NSS and MIS. These changes were seen in parallel with lower postoperative mortality and improved longer‐term survival, although these findings may also reflect temporal changes in stage case‐mix and the increasing use of effective non‐surgical treatments.

## Conflict of Interest

None.

**Abbreviations**
AAPC: average annual percent change
HES: Hospital Episode Statistics
ICD10: International Statistical Classification of Diseases and Related Health Problems 10th revision
MIS: minimally invasive surgery
NCDR: National Cancer Data Repository
NSS: nephron‐sparing surgery
ONS: Office for National Statistics

## Supporting information


**Table S1** Data fields and their source.

**Table S2** ICD10 and OPCS4 codes used in the study.

**Table S3** Patient and tumour characteristics of those treated with nephrectomy in England between 2000 and 2010.

**Table S4** Number of nephrectomies and deaths within 30 and 90 days, and in‐hospital by age at operation.
